# Factors associated with pelvic organ prolapse: case-control study in two hospitals of Bon-Berger and Saint Georges of the city of Kananga in the Democratic Republic of the Congo

**DOI:** 10.11604/pamj.2024.48.76.43545

**Published:** 2024-06-27

**Authors:** Antoine Tshimbundu Kayembe, Patrick Kahindo Muyayalo, Andy Mbangama Muela, Rahma Raschid Tozin

**Affiliations:** 1 Department of Gynaecology and Obstetrics, Faculty of Medicine, University Notre-Dame of Kasayi, Central Kasaï, Democratic Republic of the Congo,; 2 Service of Gynecology, Saint Georges Hospital of Katoka, Kananga, Central Kasaï, Democratic Republic of the Congo,; 3 Service of Gynecology, Bon-Berger Hospital of Tshikaji, Kananga, Central Kasaï, Democratic Republic of the Congo,; 4 Department of Gynaecology and Obstetrics, Faculty of Medicine, University of Kinshasa, Kinshasa, Democratic Republic of the Congo

**Keywords:** Factors associated, pelvic organs prolapse, Bon-Berger Hospital, Saint Georges Hospital, Kananga, DR Congo

## Abstract

**Introduction:**

pelvic organ prolapse is a disease or disorder of the pelvic floor that can both worsen or regress, especially in the postpartum period. It carries a high risk of recurrence after surgical treatment. The objective of this study is to identify the factors associated with pelvic organ prolapse in the two hospitals of Bon-Berger and Saint-Georges in the town of Kananga in the Democratic Republic of Congo.

**Methods:**

this is a case-control study that is carried out on the medical records of 134 patients admitted to the gynecology departments of the Bon-Berger Hospitals of Tshikaji and Saint Georges of Katoka, from January 1^st^ to July 31^st^, 2023 and based on non-probability convenience sampling for case selection. The ANOVA test, Chi-test and logistic regression with adjustment are used in the statistical analyses.

**Results:**

the factors associated with the occurrence of pelvic organs prolapse are heavy physical work (aOR: 4.031, 95% CI: 2.760-9.212; p: 0.004), malnutrition in the form of BMI less than 18.5 (aOR: 2.550, 95% CI: 1.360-5.840; p: 0.023), multiparity (aOR: 1.520, 95% CI: 1.234-4.320; p: 0.015), vaginal delivery (aOR: 3.020, 95% CI: 0.063-14.470; 0.002), fetal macrosomia (aOR: 4.290, 95% CI: 3.320-5.550; p: 0.032), pelvic tears (aOR: 2.910, 95% CI: 2.090-5.930, p: 0.006) and menopause (aOR: 3.110, 95% CI: 1.040-9.250, p: 0.001).

**Conclusion:**

these results can serve as a basis for screening women at high risk of suffering from pelvic organ prolapse during gynecological and obstetrical consultations and for in-depth studies seeking the matrix metalloproteinases associated with pelvic organ prolapse to improve its treatment in hospitals of our town of Kananga.

## Introduction

Pelvic organ prolapse is defined as a permanent or strained protrusion into or outside the vaginal canal, vulvar opening, and pelvic organs such as the uterus, bladder, rectum and small intestine [1,2]. Although it is not fatal, genital prolapse has a significant impact on the quality of life of patients and leads to serious social problems including loss of self-esteem [1-7]. According to the WHO, the pelvic organ prolapse affects around 50% of women who have given birth and its lifetime prevalence varies from 20 to 50%; which is one of the most common reasons for gynecological surgery [2-5]. Previous studies have estimated that a woman's lifetime risk of having at least one surgery for pelvic organ prolapse and urinary incontinence is 11.1%, with a 10-year reoperation rate of 17% [2,8,9].

Pelvic organ prolapse is a major cause of morbidity among women in both high- and low-income countries. The prevalence of pelvic organ prolapse varies from 2.90 to 97.70% worldwide depending on the study methods used. It is estimated from 2.90 to 11.40% when the method used is a symptom questionnaire [10-19] and from 31.80 to 97.70% when a clinical examination is carried out with pelvic organ prolapse quantification (POPQ) [8,20-28]. The global prevalence of pelvic organ prolapse has been reported to be around 9.00% and this figure is however estimated to be closer to 20.00% in low-income countries [29]. In sub-Saharan Africa, studies conducted in Ghana, Gambia, Ethiopia and Tanzania have reported prevalence rates ranging from 12 to 65.00% [30-33]. This prevalence is 24.12% in the town of Kananga in the Democratic Republic of Congo [34].

The risk factors involved in the occurrence of pelvic organs prolapse are known and divided into the following two groups: modifiable risk factors such as obesity, malnutrition, vaginal delivery, parity, smoking, fetal macrosomia, perineal tears, carrying heavy objects, low socio-economic level [1,35-39], anemia and situations of oxidative stress and cellular apoptosis in connective tissues [40-43]; and non-modifiable risk factors such as age, race including white race, menopause, chronic obstructive pulmonary disease (COPD), spinal curvature abnormalities (thoracic kyphosis, loss of lumbar lordosis), history family and personal genital prolapse, previous pelvic surgeries (hysterectomy) and chronic constipation [1,35-39,44,45].

The lack of data on factors associated with pelvic organ prolapse in our town of Kananga justifies the present study, the objective of which is to identify factors associated with pelvic organ prolapse in the two hospitals of Bon-Berger and Saint-Georges in the town of Kananga in the Democratic Republic of Congo.

## Methods

**Study design and setting:** this is a case-control study in which the cases consist of patients suffering from pelvic organ prolapse and the controls consist of patients suffering from other gynecological pathologies recorded during the mass campaign organized in the gynecological departments of two General reference hospitals from the city of Kananga: Bon-Berger Hospital in Tshikaji and Saint George Hospital in Katoka, from January 1^st^ to July 31^st^, 2023. These two hospitals were chosen because of the presence of trained and experienced medical staff, the high attendance of patients suffering from pelvic organ prolapse, and more or less free treatment of pelvic organ prolapse through the various mass campaigns in the fistula cure account. Therefore, these 2 hospitals constitute references for the treatment of pelvic organ prolapse in the city of Kananga.

**Study population:** we used the medical records of patients aged between 30 and 79 years who suffered from genital prolapse (cases) and other gynecological pathologies (controls) and were treated during the mass campaign in the gynecology departments of hospitals of Bon-Berger of Tshikaji and Saint George of Katoka in the town of Kananga from January 1^st^ to July 31^st^, 2023, and matched according to age. Our sampling is non-probabilistic for convenience. The following criteria allowed us to include the patients in the study: patients aged between 30 and 79 years, suffering from genital prolapse, treated during the mass campaign in the gynecology departments of hospitals in Bon-Berger of Tshikaji and Saint George of Katoka in the town of Kananga from January 1^st^ to July 31^st^, 2023 and whose medical file was complete. Patients who did not meet these inclusion criteria and incomplete medical records (those containing less than 50% of variables studied) or were not found were excluded. Our sample size was calculated using the following formula:


$$n\geq \frac{\left(1+\frac{1}{c}\right){\left(Z_{\alpha }+Z_{1-\beta }\right)}^{2}p\left(1-p\right)}{{\left(p_{0}-p_{1}\right)}^{2}}$$

and T= n x c [36,37,39] Where:

n = sample size

P_0_ = Controls proportion (0.0116)

P_1_= Cases proportion (0.105)


$$p_{1}=\frac{p_{0}xOR}{1+p_{0}\left(OR-1\right)};$$

P= proportion in the two groups (0.058)


$$p=\frac{p_{1}+cp_{0}}{1+c}$$

c = number of controls by cases (c=1)

T= number of controls

Zα = value of Z for the risk of the first kind (1.645)

α = the risk of type I error (0.05)

Z_1-β_= value of Z corresponding to a surface equal to the power of the test (1-β). The latter constitutes the probability of finding a significant difference (1.282). (1-β) = the desired power (0.9).

OR = minimum OR that we set for the study to be of public health interest and estimated at 10 based on studies on the Visco and Yuan model [46].

The calculated sample size is greater than 107 cases. One control will be matched to one case (c=1) and the matching criterion is the age of the patients because the latter is a confounding factor and meets the matching criteria.

We excluded all medical records not found and those containing less than 50% of the variables studied. One hundred and thirty-four cases and 134 controls were included and 4 cases were excluded because the files were not found (i.e. a total of 138 cases of pelvic organ prolapse recorded).

**Data collection:** data were collected from registers of gynecology departments, those of the operating room, medical files of patients in gynecology departments of two hospitals and the data collection record. The variables of our study are: patient age, weight, height, BMI, parity, pelvic surgery, vaginal deliveries and their number, fetal macrosomia, pelvic tears, menopause, family history of prolapse genital prolapse, personal history of pelvic organs prolapse, smoking and spinal abnormalities. The data collection was done as follows: we first identified the names of patients who had suffered from pelvic organ prolapse in the operating room and gynecology registers, then searched medical records based on the names of identified patients and finally transcribed the data from the medical records of these patients identified in the data collection sheet.

### Definitions

***Parity:*** number of pregnancies that reached 28 weeks of amenorrhea in a woman [39].

***Primiparity:*** presence or history of pregnancy that reached 28 weeks of amenorrhea in a woman [39].

***Pauciparity:*** the presence of two or three pregnancies that reached 28 weeks of amenorrhea in a woman [39].

***Multiparity:*** the story of more than four pregnancies reaching 28 weeks of amenorrhea in one woman [39].

***Body mass index (BMI):*** ratio of weight expressed in kilograms and height in meters squared [39].

***Malnutrition:*** this corresponds to a body weight of less than 50kg or a body mass index of less than 18.5kg/m^2^ [39].

**Statistical analysis:** data were analyzed using Statistical Package for Social Sciences (SPSS) software version 29. The ANOVA test was used to perform the intergroup comparison of means, the chi-square test to perform the intergroup comparison of proportions, the univariable logistic regression to evaluate the strength of association between risk factors and the occurrence of pelvic organs prolapse and multivariable logistic regression to identify the most determining factors of pelvic organs prolapse. The statistical significance threshold for our results is set at the value of p < 0.05. The variables that were statistically significantly associated with the occurrence of pelvic organ prolapse in the Anova test and chi-square test were included in the univariable model while those which were significantly associated with the occurrence of pelvic organ prolapse in the univariable model were included in the multivariable model.

**Ethical considerations:** principles of medical ethics and documentary studies rules were respected; data were collected confidentially and treated anonymously. Our study obtained authorization from the Ethics Committee of the Kinshasa Health School and the local committee of different hospitals. The reference number of the approval by the Ethics Committee is N°ESP/CE/19/2023.

## Results

The risk factors whose proportion was found to be higher, in a statistically significant manner, in the group of cases compared to that of the controls are heavy physical work, malnutrition 1 with body weight <50Kg, malnutrition 2 with BMI <18.50Kg/m^2^, multiparity, vaginal delivery, number of vaginal deliveries ≥4, fetal macrosomia, pelvic tears and menopause (Table 1).

**Table 1:** factors associated with pelvic organ prolapse

Risk factors	Case		Witnesses		Total		P-value
Age over 40 years	120	89.60%	124	92.50%	244	91.00%	0.392
Heavy physical labor and agriculture	113	84.30%	56	41.80%	169	63.10%	**<0.001**
Weight greater than 90 kg	0	0.00%	0	0,00%	0	0.00%	-
Malnutrition 1 (Weight <50Kg)	51	38.10%	2	2.20%	54	20.10%	**<0.001**
Multiparity	130	97.00%	14	10.40%	144	53.70	**<0.001**
Malnutrition 2 (BMI <18.5)	51	38.10%	0	0.00%	51	19.00%	**<0.001**
Obesity (BMI≥30)	0	0.00%	0	0.00%	0	0.00%	**-**
Anterior pelvic surgery	6	4.50%	12	9.00%	12	4.60%	0.064
Vaginal delivery	128	95.50%	30	22.40%	158	59.00	**<0.001**
Number of vaginal deliveries ≥4	130	97.40%	14	10.40	144	53.70	**<0.001**
Fetal macrosomia	92	68.70%	6	4.50%	98	36.60%	**<0.001**
Pelvic tear	100	74.60%	5	3.70%	105	39.20%	**<0.001**
Menopause	104	77.40%	74	55.20%	178	66.40%	**<0.001**
Family history of pelvic organ prolapse	0	0.00%	0	0.00%	0	0.00%	-
Personal history of pelvic organ prolapses	0	0.00%	0	0.00%	0	0.00%	-
Smoking	0	0.00%	0	0.00%	0	0.00%	-
Spinal anomaly	0	0.00%	0	0.00%	0	0.00%	-

Univariable analysis allowed us to note a significant association between the occurrence of pelvic organs prolapse and the following risk factors: heavy physical work, malnutrition in the form of body weight less than 50Kg and BMI less than 18.5, multiparity, vaginal delivery, number of vaginal deliveries greater than or equal to 4, fetal macrosomia, pelvic tear and menopause in our environment (Table 2).

**Table 2:** factors independently associated with pelvic organ prolapse

Risk factors	Univariable analysis			Multivariable analysis		
	aOR	CI 95%	P-Value	aOR	CI 95%	P-Value
Heavy physical labor and agriculture	7.495	4.202-13.367	<0.001	4.031	2.760-9.212	0.004
Malnutrition 1 (Weight <50Kg)	2.018	2.002- 2.136	<0.001	1.260	0.040-4.980	0.110
Malnutrition 2 (BMI<18.5)	2.683	0.811-8.876	<0.001	2.550	1.360-5.840	0.023
Multiparity (Parity ≥ 4)	2.004	1.001-5.011	<0.001	1.520	1.234-4.320	0.015
Vaginal delivery	7.956	2.653-18.450	<0.001	3.020	0.063-14.470	0.002
Number of vaginal deliveries ≥4	2.004	1.001-5.011	<0.001	2.510	2.250-4.910	0.998
Fetal macrosomia	4.730	1.068-11.521	<0.001	4.290	3.320-5.550	0.032
Pelvic tear	7.882	2.639-20.058	<0.001	2.910	2.090-5.930	0.006
Menopause	2.811	1.654-4.775	<0.001	3.110	1.040-9.250	0.001

According to multivariable analysis, heavy physical work (aOR: 4.031, 95% CI: 2.760-9.212; p: 0.004), malnutrition in the form of BMI less than 18.5 (aOR: 2.550, 95% CI: 1.360-5.840; p: 0.023), multiparity (aOR: 1.520, 95% CI: 1.234-4.320; p: 0.015), vaginal delivery (aOR: 3.020, 95% CI: 0.063-14.470; 0.002), fetal macrosomia (aOR: 4.290, 95% CI: 3.320-5.550; p: 0.032), pelvic tears (aOR: 2.910, 95% CI: 2.090-5.930, p: 0.006) and menopause (aOR: 3.110, 95% CI: 1.040-9.250, p: 0.001) are the independent factors associated with pelvic organs prolapse in the town of Kananga (Table 2).

## Discussion

This study aimed to identify factors associated with pelvic organs prolapse in the two hospitals of Bon-Berger and Saint-Georges in the town of Kananga in the Democratic Republic of Congo and recorded as non-molecular factors associated with pelvic organ prolapse: heavy physical work, malnutrition in the form of BMI less than 18.5, multiparity, vaginal delivery, fetal macrosomia, pelvic tears and menopause.

Heavy physical labor in the form of heavy lifting and agriculture is a factor associated with pelvic organ prolapse in our case series. It significantly increases the risk of pelvic organ prolapse occurring by 7.5. Our observations corroborate those of Masenga *et al*. in Tanzania [33], by Megabiaw *et al*. in Ethiopia [32], Scherf *et al*. in Gambia [30] and Wusu-Ansah *et al*. in Ghana [31]. The relationship between light physical work and the occurrence of pelvic organ prolapse is based on the increase, through this work, in intra-abdominal pressure, reaching 10.0±0.6 mmHg, at the basis of the loss of organs outside the pelvis and ligamentous lesions activate matrix metalloproteinases and collagen degradation [38,47,48].

Malnutrition is also a factor associated with pelvic organ prolapse in our city and significantly increases the risk of pelvic organ prolapse occurring by 2 in the form of body weight less than 50Kg and by 3 in the form of BMI less than 18.50Kg/m^2^. Our results are consistent with those of many other authors in the literature [29,30,32,33]. The occurrence of pelvic organ prolapse in malnourished women is secondary to the stimulation of massive proteolysis with a view to gluconeogenesis, this proteolysis also concerns collagen, the degradation of which leads to the loss of collagen at the pelvic ligament level, ligamentous hyperlaxity and the decline resistance to ligament traction, sources of pelvic organs prolapse [48].

Multiparity (parity ≥4) is also a factor associated with pelvic organ prolapse. It significantly increases the risk of pelvic organ prolapse occurring by 2. Our finding is in agreement, not only with that of Tshimbundu *et al*. in Kinshasa who showed that parity ≥4 is a risk factor for pelvic organ prolapse [39], but also with that of many other authors [30,36,37,49,50]. The occurrence of pelvic organs prolapse in pares ≥4 is due to the increase, linked to this parity ≥4, in the risk of pudendal nerve damage (stretching and compression) and direct trauma to the pelvic floor muscles (pubococcygeus, levator ani and anal sphincter) [47,51,52]. This leads to the defect of the pelvic floor, the site of stimulation of the activity of matrix metalloproteinases, the degradation of collagen and the reduction of collagen and tissue resistance as well as the source of occurrence of prolapse [48,53].

Like other studies [30,37,47,49,50,54], our study also showed that vaginal delivery, number of vaginal deliveries ≥4 and perineal tears are factors associated with pelvic organ prolapse. The risk of prolapse occurring was significantly increased by 7 in the case of vaginal deliveries, by 2 in the case of several vaginal deliveries ≥4 and by 8 in the case of perineal tears. Our results meet, on the one hand, those of Kayembe *et al*. in Kinshasa [39] and on the other hand those of many other authors in the literature [36,47,49]. The mechanism of occurrence of pelvic organs prolapse in cases of vaginal delivery and perineal tears is based on the increased risk of pudendal nerve damage and deterioration of the posterior level thanks to damage to the central fibrous core of the perineum and anal sphincters. These lesions activate matrix metalloproteinases to cause local collagen degradation, leading to the reduction of collagen which weakens the pelvic floor, the source of pelvic organ prolapse [47,48,51-53].

Fetal macrosomia is a factor associated with pelvic organ prolapse and increases the risk of pelvic organ prolapse occurring by 5 in our city of Kananga. Our results agree on the one hand with those of Thubert *et al*. in Kinshasa [38] and on the other hand those of many other authors in the literature where fetal macrosomia was also significantly associated with the occurrence of pelvic organ prolapse [30,36-38,47,49,50,52]. The occurrence of pelvic organ prolapse and fetal macrosomia are linked by the severity of pelvic floor lesions, secondary to the delivery of macrosomal newborns [38,47]. These lesions also stimulate the activity of matrix metalloproteinases which are the basis of collagen degradation, collagen reduction and tissue resistance, a source of pelvic organ prolapse [47,48,51-53].

Menopause is a factor associated with pelvic organ prolapse and significantly triples the risk of prolapse occurring. Our results are similar not only to those of Kayembe *et al*. in Kinshasa [39] but also to those of many authors in literature [30,47,50,54]. The role of menopause in the occurrence of pelvic organs prolapse is based on post-menopausal estrogen insufficiency which leads to changes in vaginal trophism, tissue cellularity and collagen metabolism (by stimulating the degradation of collagen by matrix metalloproteinases at the basis of collagen reduction) [47,48,55,56]. Estrogen receptors have been identified in the bladder trigone, urethra, vaginal mucosa, perineal tendinous arch, uterosacral ligaments and levator ani [47,52,55,56]. Hence the reduction in estrogen intake leads to atrophy of all of these tissues, responsible for the weakness of the pelvic floor which is the basis of the occurrence of pelvic organs prolapse [47,52,55,56].

The independent factors associated with pelvic organ prolapse in our environment are heavy physical work, malnutrition (BMI <18.5), multiparity, vaginal delivery of fetal macrosomia, complicated by pelvic tears and menopause. Our results corroborate those of other authors in the literature [30,36,38,47,49-52,55,56]. The profile of the patient suffering from pelvic organs prolapse in the town of Kananga is a malnourished, multiparous menopause with a history of vaginal delivery of a macrosome baby, complicated by perineal tears and performing heavy physical work while that of the patient suffering from pelvic organs prolapse in Kinshasa is an obese menopausal woman with a history of vaginal delivery of macrosomia, complicated by perineal tear [39]. The difference between the two profiles in the same country (DR Congo) can be explained by the fact that most patients in the town of Kananga come from surrounding rural areas where they are subjected to heavy physical work (country, etc.) for survival and that the town of Kananga is one of the areas where the deadly Kamuina-nsapu conflicts took place (commonly called the “Kamuina-nsapu phenomenon”) in 2017 with their consequences including famine and malnutrition on the local population who are struggling to survive or to emerge despite the multiple local recovery plans organized by the national government and non-governmental organizations. Hence the need to monitor and strengthen these recovery plans already established. The consideration of multiparity as a criterion for matching controls to cases in the Kinshasa study made it disappear from the profile of women suffering from pelvic organ prolapse in Kinshasa after the calculation of the multivariate logistic regression [39]. This was not the case in this study.

Our data can serve not only as a basis for screening women at high risk of suffering from pelvic organs prolapse during gynecological and obstetrical consultations to improve treatment but also as a base for more in-depth studies seeking the molecular factors associated with pelvic organs prolapse such as matrix metalloproteinases associated with pelvic organ prolapse in our town of Kananga because according to literature, all our factors associated with pelvic organs prolapse (heavy physical work, malnutrition in the form of BMI less than 18.5, multiparity, vaginal delivery, fetal macrosomia, pelvic tears and menopause) activate or stimulate the activity of matrix metalloproteinases which degrade the collagens at the base of decrease in collagen content at the pelvic level, source of pelvic ligament hyperlaxity and of pelvic organs prolapse [40,48,52,53]. In short, all our factors above would go through the increase in matrix metalloproteinases to cause pelvic organ prolapse.

The weakness of our study is the failure to take into account molecular factors associated with the occurrence of pelvic organ prolapse and its strength is to be the first study of risk factors for pelvic organ prolapse in hospital environments in the city of Kananga in the Democratic Republic of the Congo.

## Conclusion

Factors associated with the occurrence of pelvic organ prolapse are heavy physical work, malnutrition (BMI <18.5), multiparity, vaginal delivery, fetal macrosomia, pelvic tears and menopause. Our results can serve not only as a basis for screening women at high risk of suffering from pelvic organ prolapse during gynecological and obstetrical consultations but also as a basis for more in-depth studies seeking the matrix metalloproteinases associated with pelvic organ prolapse to improve its management in our town of Kananga.

### What is known about this topic

- *The pelvic organ prolapse is a dynamic disease that can worsen or recede above all in pregnant women during the postpartum period;*
- *It comprises a great recurrence risk after surgical treatment and it causes several troubles (urinary, digestive and genital) that hamper the quality of life of patients;*
- *The lack of data on factors associated with pelvic organ prolapse in hospitals in the city of Kananga, in the DR Congo*.

### What this study adds

- *Factors associated with pelvic organ prolapse are heavy physical work, malnutrition (BMI<18.5), multiparity, vaginal delivery, fetal macrosomia, pelvic tears and menopause in our town;*
- *Our results can be used in the screening of women at high risk of suffering from pelvic organ prolapse during gynecological consultations in Kananga hospitals;*
- *Our results can serve as a basis for in-depth studies seeking the matrix metalloproteinases associated with pelvic organ prolapse to improve its management in our city*.
